# Diffusive Promotion by Velocity Gradient of Cytoplasmic Streaming (CPS) in *Nitella* Internodal Cells

**DOI:** 10.1371/journal.pone.0144938

**Published:** 2015-12-22

**Authors:** Kenji Kikuchi, Osamu Mochizuki

**Affiliations:** 1 Depertment of Bioengineering and Robotics, Graduated School of Engineering, Tohoku University, Sendai, Japan; 2 Depertment of Biomedical Engineering, Toyo University, Kawagoe, Japan; A*STAR Bioinformatics Institute, SINGAPORE

## Abstract

Cytoplasmic streaming (CPS) is well known to assist the movement of nutrients, organelles and genetic material by transporting all of the cytoplasmic contents of a cell. CPS is generated by motility organelles that are driven by motor proteins near a membrane surface, where the CPS has been found to have a flat velocity profile in the flow field according to the sliding theory. There is a consistent mixing of contents inside the cell by CPS if the velocity gradient profile is flattened, which is not assisted by advection diffusion but is only supported by Brownian diffusion. Although the precise flow structure of the cytoplasm has an important role for cellular metabolism, the hydrodynamic mechanism of its convection has not been clarified. We conducted an experiment to visualise the flow of cytoplasm in *Nitella* cells by injecting tracer fluorescent nanoparticles and using a flow visualisation system in order to understand how the flow profile affects their metabolic system. We determined that the velocity field in the cytosol has an obvious velocity gradient, not a flattened gradient, which suggests that the gradient assists cytosolic mixing by Taylor–Aris dispersion more than by Brownian diffusion.

## Introduction

Cytoplasmic streaming (CPS) was first discovered by Corti (1774) [1] as a mechanism of cytosis in the green algae *Nitella* and *Chara* following the development of a handmade optical microscope. Corti discovered active fluid motion in the cell, which was initially termed ‘protoplasmic streaming’. In a plant cell, CPS is considered very important for the transport of water, nutrients and ions related to metabolism [2]. For over 20 decades, however, the mechanism of CPS has been veiled in biological mystery. There are still many uncertainties about the mechanism of CPS, particularly with respect to plant physiology and hydrodynamics. The literature about CPS includes excellent reviews that cover numerous experimental, theoretical and numerical aspects [2–5]. The motility of CPS has been hypothesised to be based on the sol-gel layer on the cell wall with slip motion, which is the ‘sliding theory’ of Kamiya and Kuroda (1956) [6]. They mentioned that the slip layer has a flattened velocity distribution, although the vacuole, which is the biggest organelle located in the centre of the cell, has a sigmoidal velocity profile. Does the velocity in the cytosol help to mix or disperse contents even if its velocity gradient is flattened? We investigated the hydrodynamic effect on dispersion in the cytosol using a microscopic flow visualisation technique.

The driving force of CPS is conducted by motor proteins in plant cells, namely actin filaments and myosin. These motor proteins can be found in several plant cells [7–9] and they seem to generate a stream of CPS using the energy of ATP, hence it is important to know where these motor proteins occur in the cell. The filaments, which consist of about 100 actin filaments, occur as a helical bundle on the cell wall for biological protein actuator working with an organelle coated with the motor protein myosin and have a series of arrowhead-like structures connecting actin monomers by reversible immobilisation. A specific myosin was also found in plant cells [10, 11]. Recently, Ueda et al. (2010) [12] identified the myosin that adheres to the endoplasmic reticulum (ER) in a plant cell. In the case of *Nitella* and *Chara*, ER was recognised on the parallel bundles of actin filaments at the interface with stationary cortical cytoplasm, which was determined by using fast-freezing electron microscopy [13]. The sliding of ER, which occurs at approximately 50–60 μm s^−1^, was visualised in a buffer with ATP and dissociated cytoplasm, thus the motive force was considered to be generated by the cytoplasm.

CPS has been experimentally observed inside *Nitella* and *Chara* by numerous researchers. However, few have measured the detailed flow distribution in the cytosol even though the cytosol plays a key role to support life by making proteins, nutrients, ATP and genetic materials. This flowing phenomenon was first observed by tracking the visible amorphous particles in motion within *Nitella* and *Chara*. Ewart (1903) [14] drew schematic figures of the velocity distribution, which has a higher speed near the wall than in the centre of the cell, according to observations with optical microscopy. Kamiya and Kuroda (1956) [6] measured the velocity distribution of CPS in *Nitella* by using a microscope with cinematographic analysis. By using two-dimensional recorded images with optical microscopy, they suggested that the speed of cytoplasm (near the wall of the sol layer) would be flattened and that cell sap, which is an organelle located at the centre of the cell, has a sigmoidal curved distribution. Mustacich and Ware (1974) [15] measured the CPS using a laser-doppler scattering system with photo-bleaching of chloroplasts as an observing window. In recent years, Meent et al. (2010) [16] measured the three-dimensional velocity distribution of the flow of cell sap in *Nitella* using a magnetic resonance technique. Although the flow near the cell wall, which is a gel-sol layer called cytosol, has a very important role for metabolism in a plant cell, it is not yet fully understood due to lack of optical spatial resolution for microscopic observation. To understand plant metabolism according to the mechanism of transport, it is necessary to visualise the flow of CPS that precisely controls plant physiology.

In this paper, we performed a flow visualisation of CPS in a *Nitella* cell by particle tracking velocimetry (PTV) with microinjection of nano-scale tracers into the cell. We aimed to clarify the flow gradient from the wall with motile organelles to sap in the centre of the cell in order to investigate the diffusive contribution of the flow inside. Though there is an invasive visualization technique by using transfected cell expressing organelles, especially peroxisome marker into a fungal hypha by Pieuchot (2015) [17], we employed the low invasive flow visualization technique using injection of tracer nano-particles into vacuole and cytosol individually for comparison between both of the internal flow. These methods were conducted precisely by measuring *in vivo* the detailed velocity distribution of CPS using confocal microscopic observations of a plant cell, and by considering the physiological reactions in response to mechanical stimulations. The flow at the cytosol shows an obvious velocity gradient, not a flattened gradient, suggesting that the gradient assists cytosolic mixing by advection diffusion more than by Brownian diffusion.

## Materials and Methods

### Nitella plant cells

*Nitella flexilis* has giant internodal cells that are suitable for observing cytoplasmic streaming due to their relatively large size (diameter >100 μm; length >5 cm) and fast motion of cytoplasmic streaming (>50 μm s^−1^). We cultured *N*. *flexilis* in our laboratory in a water tank with culture solution and air bubbling at 25°C ± 5°C. For photosynthesis, the fluorescent lamp was turned on for 16 h and off for 8 h each day. We cultured the main axis stem of *N*. *flexilis* to more than 50 mm in length for the present experiments.

**Fig 1:**
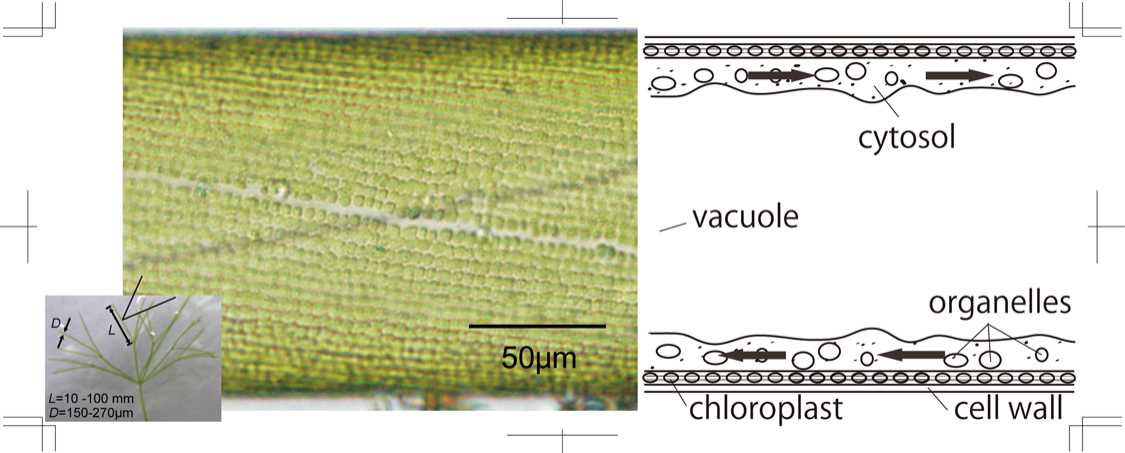
Characean algae of *Nitella flexilis* and a diagram of its internal structure. Reprinted from [3].

A diagram of the internal structure of *N*. *flexilis* is shown in Fig 1, which is a schematic figure from Shimmen (2007) [18]. The cell forms a circular cylindrical shape with a spiral arrangement of chloroplasts on the inside of the cell wall. Visible vesicles move with a one-way motion past the green organelles, or chloroplasts, fixed on the inside cell wall.

### Physiological reaction to microinjection of tracer particles

**Fig 2:**
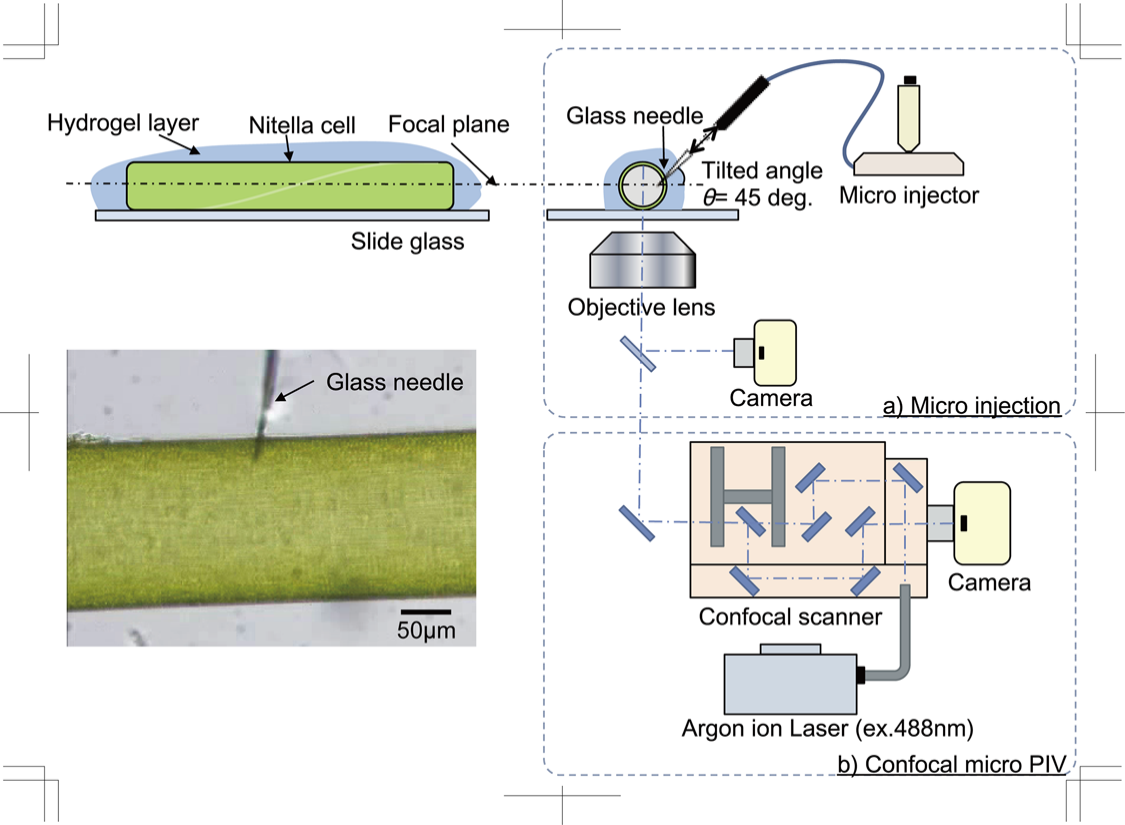
Experimental setup for visualisation of cytoplasmic streaming in a *Nitella* cell. a) Microinjection system using a glass capillary needle. b) Confocal micro PTV system.

A microinjection system of fluorescent particles was employed for flow visualisation inside the plant cell. The physiological reaction of the CPS to stimulation by a penetrating needle is necessary to confirm visualisation of the flow of microparticles after stimulation. Fig 2A) shows the microinjection system that uses a glass needle manipulated by a micromanipulator. The needle has a sharpened tip for penetrating the cell wall, which was manipulated with 1-μm minimum resolution and at 1 μm^−1^ s. The invasive stimulation to the cell was a rapid injection or removal of the needle through the cell wall surface. For microscopic observation, the cells were covered with a 10% agar gel layer, which was prepared from culture solution, to prevent the cells from moving with the stimulation. The experimental protocol for the physical stimulation using a needle is as follows:

1. Set the cell in the stimulation system and adjust the needle position manually.
2. Wait for about 5 min until the CPS of the cell is fully developed.
3. Stimulate the cell by inserting the needle.
4. Wait until the CPS of the cell is fully developed, with the needle remaining in the cell.
5. Remove the needle quickly.
6. Wait until the CPS of the cell is fully developed.

The time history of CPS during the above protocol was captured using a colour digital video image with 15 frames per second and 640 × 480 pixels resolution. This system was mounted on an inverted microscope with a 10× objective lens (NA. = 0.3). The focus position was precisely set at the centre of the cell, which was visually confirmed from a microscopic view on a projected monitor. The depth position of visible particles was identified by the brightness of the projected figure caused by light scattering in the microscope. The bright/dark figures indicated the interior/posterior position in relation to the focus plane, particularly in the focus plane where the figure shows only a clear edge. The speed of the visible organelles and vesicles were measured in response to the stimulation with a needle by manual tracking analysis using motion capture software for nine internodal cells (number of samples, N = 9).

### Measuring velocity distribution of cytoplasmic streaming using confocal micro PTV

The velocity distribution in *Nitella* cells inside the cytosol and vacuole were measured precisely using tracking particle velocimetry combined with confocal microscopy. We utilised fluorescent nanoparticles as tracers that do not have any motility in the *Nitella* cell because those surfaces have no motor proteins. Fig 2B) shows that the confocal μPTV (microparticle tracking velocimetry) system for *in vivo* visualisation of CPS in a *Nitella* internodal cell. This system consists of a confocal scanner (CSU22; Yokogawa, Japan) with an inverted microscope (IX71; Olympus, Japan) connected to an illuminating CW argon ion laser and a sensitive 12 bit mono colour CCD digital camera. For the precise fluorescent particle observation in CPS, we employed a 40× objective lens (NA. = 0.65). We used the camera to record the flow in the cell sap and cytoplasm when they were individually injected with nano tracer particles, and analysed the images using image analysis software ImageJ. The nano tracer particles have a diameter of 500 nm and contain a fluorescent dye inside (Fluosphere; Molecular imaging Inc., USA). The fluorescent intensities of particles emitted at the same depth position had at least 8.4% of standard deviation against mean intensity (N = 50). We calculated each actual depth position of moving particles by the individual fluorescent intensities, which related to the distances from the center of focus plane, hence the depth position was calculated with 0.27 μm/count in minimum resolution in our experiment. In the case of flow field in cytosol (~10 μm), this method could give us precise depth position in optical slice thickness (OST = 5.4 μm), which is calculated from an emitted wavelength, *λ*_em_ = 515 nm, a refractive index of water, *n*_w_ = 1.33, a numerical aperture (NA. = 0.65), a magnification of objective lens, *m* = 40, and a confocal pinhole diameter, *d*_p_ = 50 μm using an optical theory by Park et al (2004) [19]. The relationship between fluorescence intensity of tracer particle and depth position was calibrated in advance using a suitably angled flat plates gathering with nano fluorescent particle suspension (S1 Fig). In our experiment, we observed CPS motility inhibited by over-absorption of laser illumination. Continuous high-intensity laser for over 15 min was absorbed by the chloroplasts, which caused photo bleaching that inhibited CPS motility, and the chlorophyll became transparent.

## Results

### Physiological reaction in response to needle stimulation

**Fig 3:**
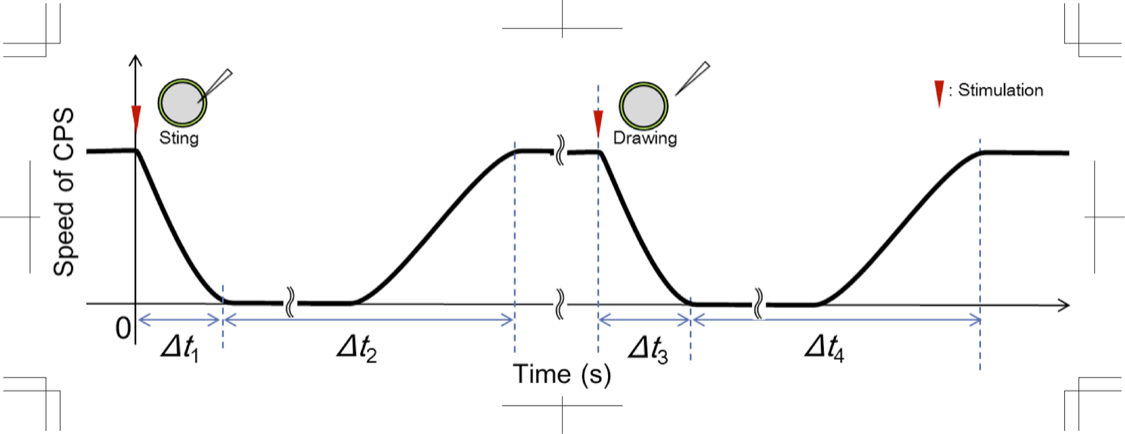
The change in speed of CPS over time in response to needle stimulation.

The needle stimulations, i.e. inserting or removing the needle, caused the CPS to immediately stop, indicating a physiological reaction, after which the speed of the CPS gradually recovered. The time history of the change of speed of CPS is shown in Fig 3.

Immediately after inserting the needle, the CPS speed decreased rapidly (S1 Video). The initial speed reached zero with *Δt*_*1*_. After the CPS stopped, it started to move again gradually and reached the initial speed at *Δt*_*2*_. At this moment the needle remained in the cell. Five minutes later the CPS recovered its initial speed, despite the needle still penetrating the cell. Next, the needle was removed quickly, and then the CPS decreased rapidly in its speed again with *Δt*_*3*_ (S2 Video). Eventually the CPS recovered, reaching the same velocity at *Δt*_*4*_ as at *Δt*_*2*_. The average diameter *d* and the average speed |*v|* of visible particles in the cell, and the time *Δt*_*1*_, *Δt*_*2*_, *Δt*_*3*_ and *Δt*_*4*_ measured for 20 particles selected at random in each of nine samples are shown in Table 1. We defined the recovery time from needle stimulation, *T*_*rc*_ *= Δt*_*3*_ + *Δt*_*4*_, as the reference time to start to record the velocity distribution on the PTV measurement. The diameter and speed of the invisible particles are in good agreement with previous studies [2, 6, 14, 16, 18, 20–21]. The attenuation times when CPS decreased from the initial speed to zero, *Δt*_*1*_ and *Δt*_*3*_, are almost the same, approximately 1.2 s, even though the stimulation was different. A previous study of mechanical and electrical stimulation to a *Nitella* internodal cell suggested that CPS also stopped momentarily just after the stimulation. A similar tendency was recognised when CPS recovered as the recovery time was approximately 2 min. There was a time lag for CPS recovery after inserting and removing the needle, hence we started to record the velocity measurement by confocal μPTV method 2 min after removing the needle, and then injected the nano tracer particles into the cell.

**Table 1:** Motility of visible particles in *Nitella* internodal cells.

Diameter	CPS	Interval time				Recovery time
*d* [μm]	$\left|\bar{v}\right|$ [μm s^-1^]	***Δt***_***1***_ [s]	***Δt***_***2***_ [s]	***Δt***_***3***_ [s]	***Δt***_***4***_ [s]	*T*_*rc*_ [s]
6.2 ± 3.2	9.4 ± 3.2	1.1 ± 0.3	116.8 ± 2.4	1.3 ± 0.7	115.8 ± 4.1	117.1± 4.8

### Observation of flow in the vacuole

**Fig 4:**
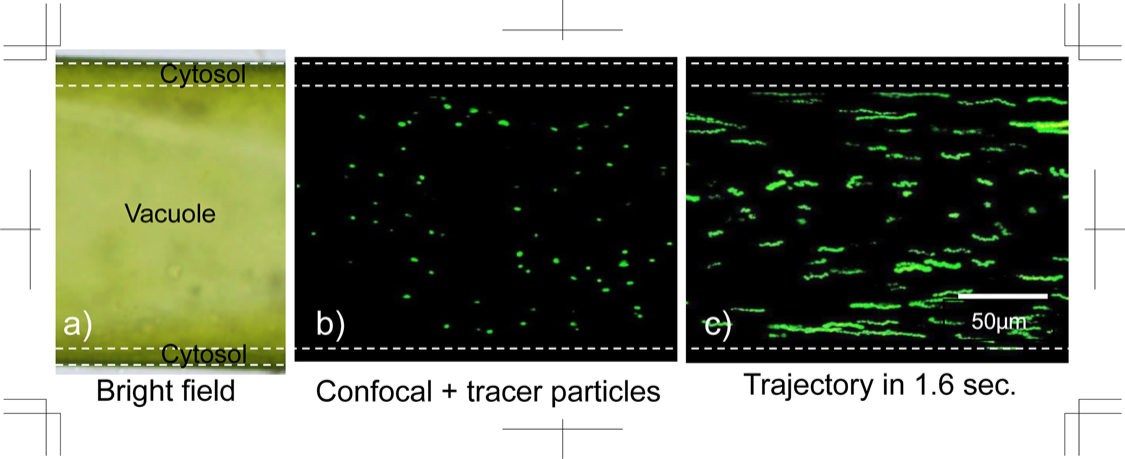
Flow visualisation in the vacuole of a *Nitella* internodal cell. a) Bright field view. b) Fluorescent particles injected into a vacuole. c) Trajectory of flow in a vacuole.

**Fig 5:**
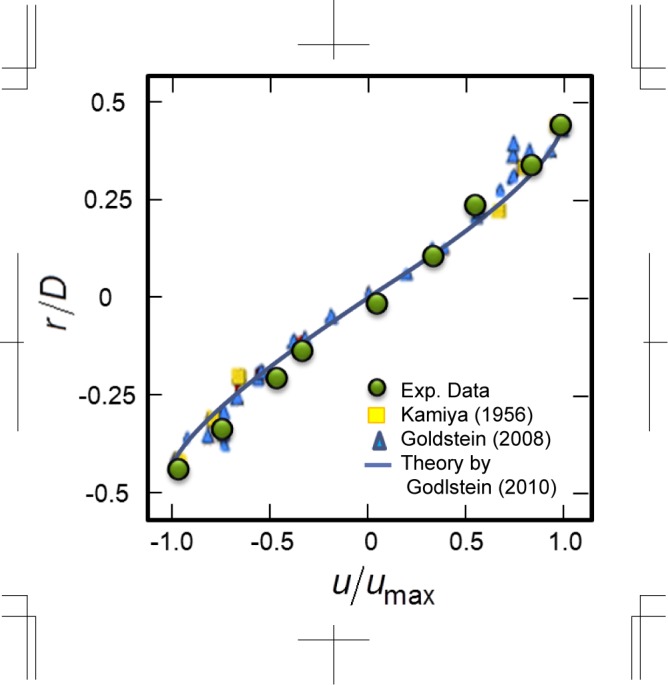
Flow distributions in vacuoles of Nitella internodal cells compared with those from previous studies.

Fig 4 shows the experimental results of flow visualisation in vacuoles of *Nitella* internodal cells by using confocal μPTV methods. The vacuole, which is surrounded by cytosol, occupies the centre of the cell as shown in Fig 2A). The fluorescent particles were injected into the vacuole using the micromanipulator (Fig 2B)), and they spread throughout the whole vacuole medium without leaking into other organelles, particularly not into the cytosol. The multiple-time superimposed images produced the trajectories of the CPS in the vacuole as a velocity profile *v*_*v*_(*r*/*D*) as shown in Fig 4C). By comparing the length of the trajectories, relatively fast velocities were found to occur near the wall rather than in the centre. The average velocity, $\left|{\bar{v}}_{v}\right|=\sum_{\left|r/d=0\right|}^{0.5}\left|v_{v}\left(r/d\right)\right|$ in the vacuole was indicated to be 9.3 ± 4.2 μm s^−1^, which is similar to the average velocity $\left|\bar{v}\right|$ = 9.4 ± 3.2 μm s^−1^ of visible particles shown in Table 1, hence the nanoparticles accurately traced the flow inside the vacuole. We measured the velocity distribution inside the vacuole and compared the results with our experimental data from previous studies (Fig 5). The flow inside a *Nitella* cell usually shows helical streaming along the fixed lines of chloroplasts on the cell wall, hence we compared the velocity profile at the cell wall, which has the most countercurrent flow. The subsequent velocity fields that were measured inside the vacuole agreed with results from the previous study, namely the CPS in a *Nitella* cell was flowing in the vacuole.

### Observation of flow in the cytosol

**Fig 6:**
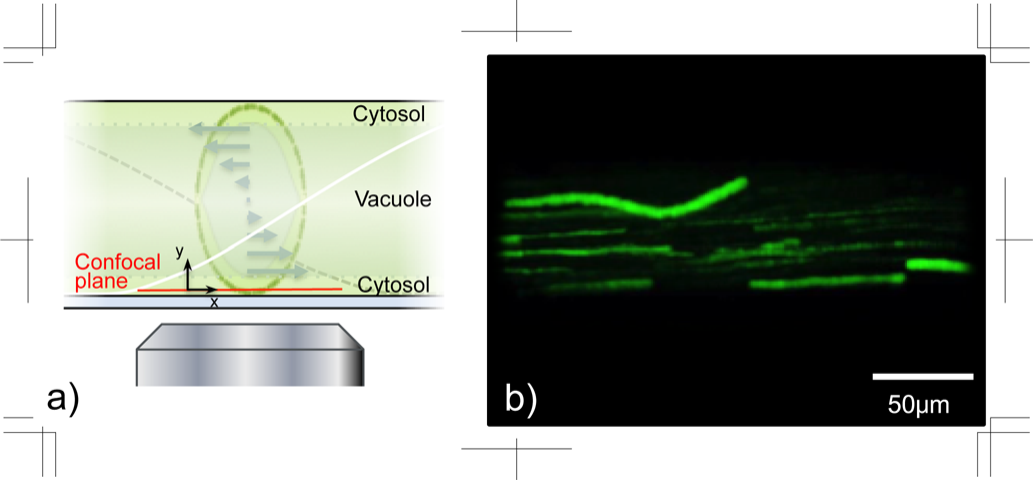
Flow visualisation in the cytosol of a *Nitella* internodal cell. a) Schematic of the observation region near the cell wall in the cytosol. b) Trajectory of flow in the cytosol in 1.6 sec.

The flow distribution of the cytosol, which gathers the cell membrane and cell sap in a narrow region, was measured using confocal microscopy. The cytosolic layer in our experimental conditions was about 10 μm or less. The nanoparticles were injected into the cytosol with precisely controlled manipulation and we visualised the flow inside the cytosol. The particles were dispersed just after the injection along with the helical flow in the cytosol (S3 Video). The focal plane was set at the bottom of the cytosolic flow, 4 μm from the cell wall (Fig 6A)). We defined the bottom of the cytosol near the cell wall by the existence of tracer particles, where the particles behaved with different and meandering trajectories (Fig 6B)). On the other hand, the tracer particles did not find between the bottom of cytosol and cell wall. We measured the flow velocity manually by tracking the particles using ImageJ, and the trajectories of the flow in the cytosol were obtained from the multiple-time superimposed images (Fig 6B)). Most particles flowed straight but the particles near the bottom behaved in a meandering trajectory. The intensity of the fluorescent particles indicated their height from the bottom of the cytosol, which is because the decay intensity is related optically to the distance from the focal plane. The axial intensity distribution has been represented theoretically based on a function of Gaussian distribution by Cole et al. (2011) [22].

**Fig 7:**
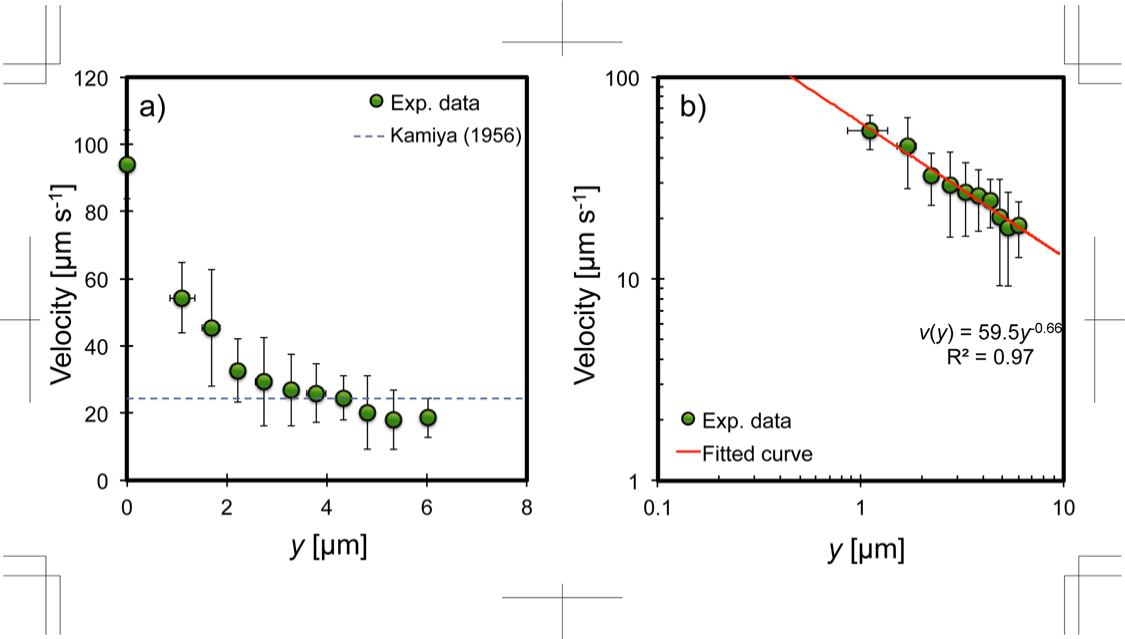
Velocity distribution of the cytosol in Nitella internodal cells. a) Linear scales. b) Logarithmic scales with a power approximation curve.

The relationship between the intensity of a fluorescent particle and its height was measured and a fitting curve was obtained according to the following equation:

$$\frac{I\left(\delta \right)}{I_{0}}=\frac{1}{\sigma \sqrt{2\pi }}\exp\left(\frac{-y^{2}}{2\sigma^{2}}\right)$$

where *I* is the local intensity; *y* is the distance from the confocal plane, namely the height position from the bottom of the cytosol; *I*_0_ is the brightest intensity near the confocal plane, so that the left term presents the normalised intensity; *σ* is the standard deviation in our experimental condition, which is 2.0, indicating good agreement with experimental data and the above Gaussian distribution. From this equation the particle position was determined from its intensity, and then the flow distribution in the cytosol was obtained, which has an explicit velocity gradient (Fig 7A)). The maximum velocity measured at the bottom of the cytosol was *v*_max_ = 94.03 ± 10.12 μm s^−1^. The velocity clearly decreased with increasing distance from the bottom of the cytosol. Since the maximum velocity in Fig 7A) was obtained at 4 μm apart from the cell wall, the large discrepancy was occurred totally approximately 6 μm apart from the cell wall. It might be caused by existing of the endoplasmic reticulum networks [14], witch move along with the actin rail, flowing with several heights. We plotted the velocity distribution on a logarithmic scale as shown in Fig 7B). The fitted curve is a power approximation curve, *v*(*y*) = 59.5*y* ^−0.66^, with R^2^ = 0.97 for the determination coefficient. Our result indicates that the velocity gradient in the cytosol clearly exists, although former researchers believed that the cytosol in *Nitella* internodal cells has a flattened velocity distribution as originally reported by Kamiya (1956) [6].

## Discussion

We performed an experiment to confirm the inhibition of CPS and to measure the recovery time of CPS after needle stimulation. Upon needle stimulation, the visible vesicles were transiently inhibited just after inserting the needle into the cell, but they recovered gradually and recovered their initial speed. This intermittent stoppage of the CPS is considered to be a physiological reaction of plants, hence similar behaviour was observed in the case of mechanical stimulation [23–25]. Shimmen (1996) [25] suggested that intermittent cessation of CPS by mechanical stimulation foccurred by triggering an action potential induced by activation of Ca^2+^ and Cl^−^ channels. Tazawa (1968) [26] had mentioned that the relationship between the driving force and CPS simultaneously occurred to induce the positive release of Ca^2+^ at the endoplasm interface after cessation of CPS by an electrical stimulus.

After an intermittent cessation, the CPS still had a creeping motion for about 1 min. If the driving force stops impulsively in Newtonian fluids, the flow should stop immediately due to its incompressibility. However, the creeping motion appeared after the driving force ceased in the CPS; thus, the fluid in the cell is behaving as a non-Newtonian fluid. Hayashi (1980) [27] performed a theoretical study for motive force and its non-Newtonian behaviour on internal cellular fluid [28], so that creeping of the visible vesicles would seem to be incurred by non-Newtonian behaviour. In our experiments, the speed of the CPS after needle stimulation gradually recovered to the initial speed after about 2 min. This recovery of CPS gives us the CPS measurements in the fully developed state after microinjection of the particles due to the flow is enough to be assumed as quasi-steady state.

We measured the precise cross-sectional velocity field of the vacuole in *Nitella* internodal cells by using confocal microscopic observation with microinjection of nano tracer particles. Our data indicate a similar velocity distribution to previous reports (Fig 5), which is a cross-sectional velocity field of helical flow, hence an apparent agreement with the theoretical value constructed by Meent (2010) [16]. The maximum velocity in the vacuole appears to be near the outside wall and reaches ~100 μm s^−1^, which is almost the same speed as found in previous reports. This suggests that the injected tracer particles work normally with no inhibition to flow in the vacuole, and thus it seems that the present microinjection technique would be of low invasiveness to *Nitella* cells.

Although it is generally accepted that the flow distribution inside the cytoplasm is flattened [6, 13, 17, 26, 27], there is an obvious velocity gradient in our observations (Figs 6 and 7). If flattened velocity fields exist in the entire cytosol as mentioned by previous researchers, based on the slip boundary, there would be no large role for mixing or transporting materials because all of the cytosolic contents would have the same translational velocity gathered by slip from the walls. Dispersion in the cytosol would then depend only on Brownian diffusion, $D_{Brown}=\frac{kT}{3\pi \mu d_{p}}=4.40\times 10^{-12}$ m^2^ s^−1^, to assist dispersion inside the cell. In the Brownian diffusion coefficient, *k* is the Boltzmann constant at 1.38 × 10^−23^ J K^−1^, *T* is the absolute temperature at 300 K, μ is the water viscosity at 9.97 × 10^−4^ Pa s, *d*_*p*_ is the particle diameter at 100 nm assuming a small vesicle size produced by endocytosis [29, 30]. This means that a protein produced in a plant cell would be transported with a transport time $t_{Brown}=\frac{h^{2}}{6D_{Brown}}$ s for a height of cytosol, *h* = 10 μm measured from our observation, which is the same scale as in a previous study [16]. The Péclet number in the cytosol, $Pe=\frac{{\bar{v}}_{c}h}{D_{Brown}}=87.4$, suggests that advection is more dominant than diffusion. Here ${\bar{v}}_{c}$ is the average velocity in the cytosol estimated as $\left|{\bar{v}}_{c}\right|=\frac{1}{h}\int_{0}^{h}v_{c}\left(y\right)dy=38.5$ μm s^−1^. There seems to be conflicting explanations about the role of CPS, which would have enough mixing effect in large plant cells; however, such a Péclet number scale possesses fully developed diffusion to the whole length of the *Nitella* internodal cell, ~1 cm, of approximately 44 days! On the other hand, dispersion in a pipe flow with a radial shear gradient, which is well known as the Taylor–Aris dispersion [31], works as a radial dispersion, and consequently it effectively provides a longitudinal diffusion as the effective dispersion coefficient [32], $D_{eff}=D_{Brown}+\gamma \frac{{\bar{v}}^{2}h^{2}}{D_{Brown}}=D_{Brown}\left(1+\gamma Pe^{2}\right)$. Here, γ is the geometric factor that depends on the flow distribution and the boundary condition in the flow field, discussed in detail in S1 Text, as being $\frac{13}{280}$, because *D*_*eff*_ = 1.57 × 10^−9^ m^2^ s^−1^ and $t_{eff}=\frac{h^{2}}{6D_{eff}}=1.06\times 10^{-2}$ s. This means that dispersion with a velocity gradient in the cytosol is 356 times faster than molecule dispersion with a flattened velocity field because of the dispersion ratio *D*_*eff*_ / *D*_*Brown*_ = 356. In fact, the fully developed dispersion to the entire *Nitella* internodal cell reduces to only 3 hours by this Taylor–Aris dispersion effect. Namely, we consider that the mixing and transport role in CPS helps to diffuse small particles or proteins in the cell by a velocity gradient in the cytosolic flow.

## Supporting Information

S1 Fig Calibration of depth position comparing with intensity of fluorescent nano particle. (TIF)

S1 Text Geometric constant of Taylor-Aris dispersion. (PDF)

S1 Video Cytoplasmic streaming stoppage by sting a needle into a cell as mechanical stimulation. (MP4)

S2 Video Cytoplasmic streaming stoppage by drawing a needle from a cell as mechanical stimulation. (MP4)

S3 Video Particle dispersion driven by inside flow in the cytosol. (MP4)

## References

[1] Corti B. Osservazioni microscopiche sulla tremella e sulla circolazione del fluido in una pianta acqua juola. 1774: G. Rocchi.

[2] Allen NS, Allen RD, Cytoplasmic streaming in green plants. Annu Rev Biophys Bioeng. 1978; 7: 497–526. 352247 10.1146/annurev.bb.07.060178.002433

[3] Kamiya N. Physical and Chemical Basis of Cytoplasmic Streaming. Annual Review of Plant Physiology. 1981; 32: 205–236.

[4] Shimmen T., Yokota E. Cytoplasmic streaming in plants. Current Opinion in Cell Biology. 2004; 16: 68–72. 15037307 10.1016/j.ceb.2003.11.009

[5] Goldstein RE, Van de Meent JW. A physical perspective on cytoplasmic streaming. Vol. 5. 2015; 20150030. 10.1098/rsfs.2015.0030 PMC4590424 26464789

[6] Kamiya N, Kuroda K. Velocity distribution of the protoplasmic streaming in Nitella cells, in Bot Mag Tokyo. 1956. pp. 544–554.

[7] Williamson RE. Act in the alga, Chara corallina. Nature. 1974; 248: 801–802. 4835552 10.1038/248801a0

[8] Palevitz BA, Ash JF, Hepler PK. Actin in the green alga, Nitella. Proc Natl Acad Sci U S A. 1974; 71: 363–366. 4592689 10.1073/pnas.71.2.363 PMC388005

[9] Staiger CJ, Schliwa M. Actin localization and function in higher plants. Protoplasma. 1987; 141: 1–12.

[10] Ma YZ, Yen LF. Actin and myosin in pea tendrils. Plant physiology. 1989; 89: 586–589. 16666586 10.1104/pp.89.2.586 PMC1055885

[11] Kato T, Tonomura Y. Identification of myosin in Nitella flexilis. J Biochem. 1977; 82: 777–782. 144121 10.1093/oxfordjournals.jbchem.a131754

[12] Ueda H, Yokota, Kutsuna E, Shimada N, Tamura T, T K Shinmen, et al Myosin-dependent endoplasmic reticulum motility and F-actin organization in plant cells. Proceedings of the National Academy of Sciences. 2010; 107: 6894–6899. 10.1073/pnas.0911482107 PMC2872430 20351265

[13] Kachar B, Reese TS. The mechanism of cytoplasmic streaming in characean algal cells: sliding of endoplasmic reticulum along actin filaments. The Journal of Cell Biology. 1988; 106: 1545–1552. 3372589 10.1083/jcb.106.5.1545 PMC2115060

[14] Ewart AJ. On the physics and physiology of protoplasmic streaming in plants, by Alfred J. Ewart. Communicated to the Royal society by Francis Gotch. With seventeen illustrations. 1903; Available: http://www.biodiversitylibrary.org/bibliography/19799.

[15] Mustacich RV, Ware BR. Observation of Protoplasmic Streaming by Laser-Light Scattering. Physical Review Letters. 1974; 33: 617–620.

[16] Van de Meent J W, Sederman AJ, Gladden LF, Goldstein RE. Measurement of cytoplasmic streaming in single plant cells by magnetic resonance velocimetry. Journal of Fluid Mechanics. 2010; 642: 5–14.

[17] Pieuchot L, Lai J, Loh RA, Leong FY, Chiam KH, Stajich J, et al Cellular Subcompartments through Cytoplasmic Streaming. Developmental Cell. 2015; 34: 410–420. doi: 10.1016/j.devcel.2015.07.017 26305593 10.1016/j.devcel.2015.07.017

[18] Shimmen T. The sliding theory of cytoplasmic streaming: fifty years of progress. J Plant Res. 2007; 120: 31–43. 17252175 10.1007/s10265-006-0061-0

[19] Park JS, Choi CK, Kihm KD. Optically sliced micro-PIV using confocal laser scanning microscopy (CLSM), Experiments in Fluids, 2004; 37105–119.

[20] Van de Meent JW, Tuval I, Goldstein RE. Nature’s Microfluidic Transporter: Rotational Cytoplasmic Streaming at High Péclet Numbers. Physical Review Letters. 2008; 101: 178102 18999789 10.1103/PhysRevLett.101.178102

[21] Mustacich RV. Observation of protoplasmic streaming by laser-light scattering. Physical review letters. 1974; 33: 617.

[22] Core RW, Jinadasa T, Brown CM. Measuring and interpreting point spread functions to determine confocal microsope resolution and ensure quality control, Nature Protcols. 2011; 6: 1929–1941. 10.1038/nprot.2011.407 22082987

[23] Tsuchiya Y, Yamazaki H, Aoki T. Steady and transient behaviors of protoplasmic streaming in Nitella internodal cell. Biophys J. 1991; 59: 249–251. 19431786 10.1016/S0006-3495(91)82215-9 PMC1281135

[24] Kaneko T, Saito C, Shimmen T, Kikuyama M. Possible Involvement of Mechanosensitive Ca2+ Channels of Plasma Membrane in Mechanoperception in Chara. Plant and Cell Physiology.2005; 46: 130–135. 15659450 10.1093/pcp/pci004

[25] Shimmen T. Studies on Mechano-Perception in Characean Cells: Development of a Monitoring Apparatus. Plant and Cell Physiology. 1996; 37: 591–597.

[26] Tazawa M. Motive force of the cytoplasmic streaming in nitella. Protoplasma. 1968; 65: 207–22. 5667678 10.1007/BF01666379

[27] Hayashi Y. Theoretical study of motive force of protoplasmic streaming in a plant cell. 1980; 85: 469–480.

[28] Allen NS. Endoplasmic filaments generate the motive force for rotational streaming in Nitella. J Cell Biol. 1974; 63: 270–87. 4608919 10.1083/jcb.63.1.270 PMC2109324

[29] Wolff K, Marenduzzo D, Cates ME. Cytoplasmic streaming in plant cells: the role of wall slip, in J R Soc Interface. 2012: England. p 1398–408. 10.1098/rsif.2011.0868 PMC3350745 22337633

[30] McMahon HT, Boucrot E. Molecular mechanism and physiological functions of clathrin-mediated endocytosis. Nature Reviews Molecular Cell Biology. 2011; 12: 517–533. doi: 10.1038/nrm3151 21779028 10.1038/nrm3151

[31] Vedel S, Hovad E, Bruus H. Time-dependent Taylor–Aris dispersion of an initial point concentration. Journal of Fluid Mechanics. 2014; 752: 107–122.

[32] Aris R. On the Dispersion of a Solute in a Fluid Flowing through a Tube. 1956.

