# Supplementary figures and images for: Diffusive Promotion by Velocity Gradient of Cytoplasmic Streaming (CPS) in Nitella Internodal Cells
Source: PLoS One. 2015 Dec 22;10(12):e0144938. doi: 10.1371/journal.pone.0144938 (PMC4690613; doi:10.1371/journal.pone.0144938)

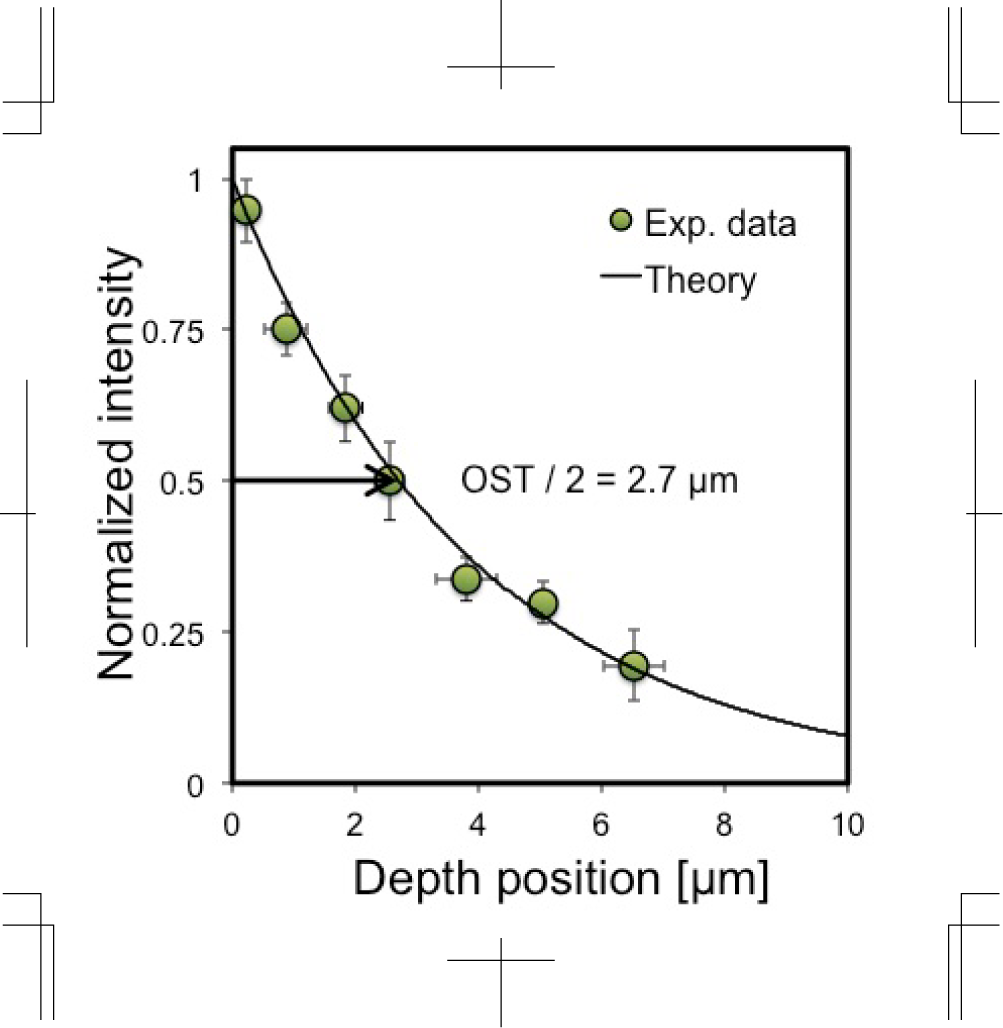

Supplement: S1 Fig — (TIF) [file pone.0144938.s001.tif]
