# Supplementary material for: Diffusive Promotion by Velocity Gradient of Cytoplasmic Streaming (CPS) in Nitella Internodal Cells
Source: PLoS One. 2015 Dec 22;10(12):e0144938. doi: 10.1371/journal.pone.0144938 (PMC4690613; doi:10.1371/journal.pone.0144938)
Supplement: S1 Text — (PDF) [file pone.0144938.s002.pdf]

Text S1. Geometric constant on Taylor–Aris dispersion

The effective dispersion coefficient is defined by Aris [29] is

$$D_{eff} = D_{Brown} + \gamma \frac{\bar{v}^2 h^2}{D_{Brown}},$$

where, D_{Brown} is the Brownian diffusion coefficient, h is the characteristic length, $\bar{v}$ is the average velocity, $\gamma = \overline{\chi\varphi}$ is the geometric factor and χ is the normalised deviation from the mean flow velocity,

$$\chi(y) = \frac{v(y)}{\bar{v}} - 1$$

where, φ is the satisfied function to

$$\left.\begin{aligned} h^2\nabla^2\varphi = -\chi, \quad & on\ R \\ \frac{\partial\varphi}{\partial n} = 0, \quad & on\ \partial R \end{aligned}\right\}$$

where, R represents cross-section, ∂R represents its boundary and $\frac{\partial}{\partial n}$ is the normal derivative.

We now calculate χ for the flow velocity field in the cytosol:

$$\begin{aligned} \chi &= \frac{\alpha\zeta^{-\frac{2}{3}}}{\int_0^1 \alpha\zeta^{-\frac{2}{3}} d\zeta} - 1 \\ &= \frac{1}{3}\zeta^{-\frac{2}{3}} - 1 \end{aligned}$$

Here $\zeta = \frac{y}{h}$, hence:

$$\left.\begin{aligned} \frac{d^2\varphi}{d^2\zeta} = 1 - \zeta^{-\frac{2}{3}}, \quad & for\ 0 < \zeta < 1 \\ \frac{\partial\varphi}{\partial n} = 0, \quad & at\ \zeta = 0, 1 \end{aligned}\right\}$$

The satisfied function φ is

$$\varphi = \frac{1}{2}\zeta - \frac{3}{4}\zeta^{\frac{4}{3}}$$

We can calculate the geometric factor γ:

$$\gamma = \int_0^1 \left(\frac{1}{3}\xi^{-\frac{2}{3}} - 1 \right) \left(\frac{1}{2}\xi - \frac{3}{4}\xi^{\frac{4}{3}} \right) d\xi$$
$$= \frac{13}{280}$$

Now we have the effective longitudinal diffusion for the flow velocity field in the cytosol:

$$D_{eff} = D_{Brown} + \frac{13}{280} \frac{\overline{v}^2 h^2}{D_{Brown}}$$
